# Altering N_2_O emissions by manipulating wheat root bacterial community

**DOI:** 10.1038/s41598-019-44124-3

**Published:** 2019-05-20

**Authors:** Alla Usyskin-Tonne, Yitzhak Hadar, Dror Minz

**Affiliations:** 1 Soil, Water and Environmental Sciences, Agricultural Research Organization, Volcani Center, Rishon LeZion, Israel; 2 Robert H. Smith Faculty of Agriculture, Food and Environment, The Hebrew University of Jerusalem, Rehovot, Israel

**Keywords:** Microbial ecology, Applied microbiology

## Abstract

Nitrous oxide (N_2_O) is a greenhouse gas and a potent ozone-depleting substance in the stratosphere. Agricultural soils are one of the main global sources of N_2_O emissions, particularly from cereal fields due to their high areal coverage. The aim of this study was to isolate N_2_O-reducing bacteria able to mitigate N_2_O emissions from the soil after inoculation. We isolated several bacteria from wheat roots that were capable of N_2_O reduction *in vitro* and studied their genetic potential and activity under different environmental conditions. Three of these isolates- all carrying the nitrous oxide reductase-encoding clade I *nosZ*, able to reduce N_2_O *in vitro*, and efficient colonizers of wheat roots- presented different N_2_O-reduction strategies when growing in the root zone, possibly due to the different conditions *in situ* and their metabolic preferences. Each isolate seemed to prefer to operate at different altered oxygen levels. Isolate AU243 (related to *Agrobacterium/Rhizobium*) could reduce both nitrate and N_2_O and operated better at lower oxygen levels. Isolate AU14 (related to *Alcaligenes faecalis*), lacking nitrate reductases, operated better under less anoxic conditions. Isolate NT128 (related to *Pseudomonas stutzeri*) caused slightly increased N_2_O emissions under both anoxic and ambient conditions. These results therefore emphasize the importance of a deep understanding of soil–plant–microbe interactions when environmental application is being considered.

## Introduction

Nitrous oxide (N_2_O) is a long-lived greenhouse gas that is ca. 300 times more active under absorbing infrared radiation than carbon dioxide per unit mass^1,2^, as well as a potent ozone-depleting substance in the stratosphere^3^. Agricultural soils are the main anthropogenic source of N_2_O emissions^4^ and their impact has escalated through the application of nitrogen-based fertilizers. The global increase in total N_2_O emissions is a continuous concern, especially from cereal production and in particular from wheat fields^5^, considering their high areal coverage (around 14 billion ha worldwide) and rising demand (www.fao.org/faostat/en/#data/QC)^6–8^. Large N_2_O emission from agricultural soils results from N_2_O accumulation during denitrification, when the first three denitrification enzymes—nitrate reductase, nitrite reductase, and nitric oxide reductase—are more active than nitrous oxide reductase, the latter considered the sole enzyme able to reduce N_2_O to nitrogen gas^9,10^. As far as we know today, the primary N_2_O producers are the denitrifying, nitrifying and methanotrophic bacteria, while only some denitrifying prokaryotes are able to reduce N_2_O to nitrogen gas^11^. Hallin and colleagues^12^ have suggested possible benefits for the microorganisms able to reduce N_2_O: (1) N_2_O acts as an electron sink, and activity of the nitrous oxide reductase-encoding gene *nosZ* sustains viability during short periods of anoxia, and (2) detoxification of nitric oxide. Many denitrifying bacteria lack the *nosZ* gene^13^ and quantification of denitrification genes in soils has shown that bacteria having *nosZ* are less abundant than those with the other denitrifying genes^12,14^. Moreover, the ability of denitrifying bacteria to reduce N_2_O depends on abiotic conditions, such as oxygen level, pH and carbon availability^15^.

Practices for mitigation of N_2_O emission from agricultural soils have included use of the nitrification inhibitor dicyandiamide, more frequent application of smaller doses of fertilizer, replacement of urea with ammonium sulfate, and biochar application^16,17^. However, very few microbiological mitigation methods involving soil inoculation of N_2_O-reducing denitrifiers have been described. Examples of these latter methods include inoculation of pelleted poultry manure with *Azoarcus*, *Niastella* and *Burkholderia* spp., and its subsequent use as a soil fertilizer, resulting in significantly lower (ca. 40–60%) N_2_O emissions compared to controls^18^. In a greenhouse study, inoculation of soil in which red clover was being grown with seven denitrifier strains affiliated with *Azospirillum* and *Herbaspirillum* significantly decreased N_2_O emissions, while similar inoculation of soil in which timothy grass was being grown did not reduce N_2_O emissions significantly^19^. In addition, field-scale experiments to examine the possibility of mitigating N_2_O emissions from soybean were conducted by inoculation of nodules with N_2_O-reducing *Bradyrhizobium diazoefficiens* mutants^20^, and with a mixed culture of indigenous symbiotic *B. diazoefficiens* strains^21^. These last two studies concluded that using efficient native strains is more effective than using mutants.

In the current study, the possibility of mitigating N_2_O emissions by inoculating wheat roots with native root-associated N_2_O-reducing bacteria was examined. Dynamics of bacterial colonization of the roots from inoculated soil was monitored by real-time PCR. N_2_O emission was measured under both anoxic (nitrogen-flushed) and ambient atmosphere, to mimic a broad spectrum of soil conditions. This novel approach to mitigating N_2_O emission from agricultural soils may provide an additional environmental tool in the fight against these harmful emissions.

## Results and Discussion

### Potential denitrification pathways in selected isolates

**Figure 1:**
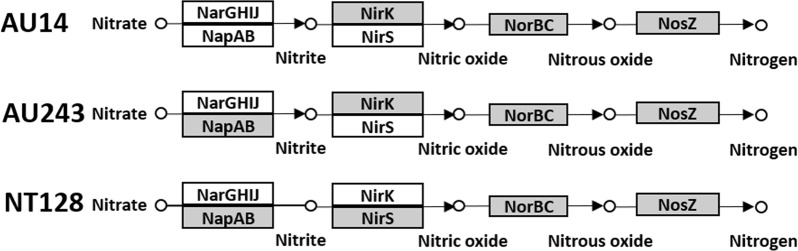
Potential metabolic denitrification pathways of N_2_O-reducing bacteria isolated from wheat roots (genes that are present in each genome are marked in gray). Nar, nitrate reductase; Nir, nitrite reductase; Nor, nitric oxide reductase; Nos, nitrous oxide reductase.

Bacteria were isolated from wheat roots based on their ability to grow under anaerobic conditions, with N_2_O as sole electron acceptor and three carbon sources (acetate, galactose and arabinose). The carbon sources in the enrichment medium were chosen to mimic wheat rhizodeposition^22^: galactose and arabinose as products of polysaccharide-degrading enzymes in the wheat root microbial community^23^, and acetate for its wide use in the cultivation of denitrifiers^24^. Out of 101 isolates tested, five showed efficient N_2_O reduction during 48 h growth from inoculation in liquid medium (2.9E6–4.8E6 ng N-N_2_O), 67 reduced N_2_O moderately (1.1E4–1.6E6 ng N-N_2_O), and 29 increased N_2_O concentration. A low percentage of N_2_O-reducing isolates was expected, based on the relatively low abundance and expression of *nosZ* in many niches, including wheat roots^23,25,26^, and its low activity^27^. After the initial screening, two isolates were selected for the current study (AU243 and AU14) based on their superior N_2_O-reducing ability, along with the previously isolated NT128, described by Tovi *et al*.^28^ Based on whole-genome sequence analysis, isolate NT128 is closely related to *P. stutzeri*^28^, isolate AU14 to *A. faecalis* and AU243 to the genus *Agrobacterium/Rhizobium*. Isolates AU243 and NT128 have all four genes required for complete denitrification^29^ and are thus potentially able to fully reduce nitrate to atmospheric nitrogen (Fig. 1). Isolate AU14, however, is lacking genes for nitrate reduction to nitrite, and is thus unable to use nitrate as the terminal electron acceptor (Fig. 1). Having clade I *nosZ* genes, all three isolates have the potential to reduce N_2_O to atmospheric nitrogen under anaerobic conditions.

### N_2_O reduction in pure culture

**Figure 2:**
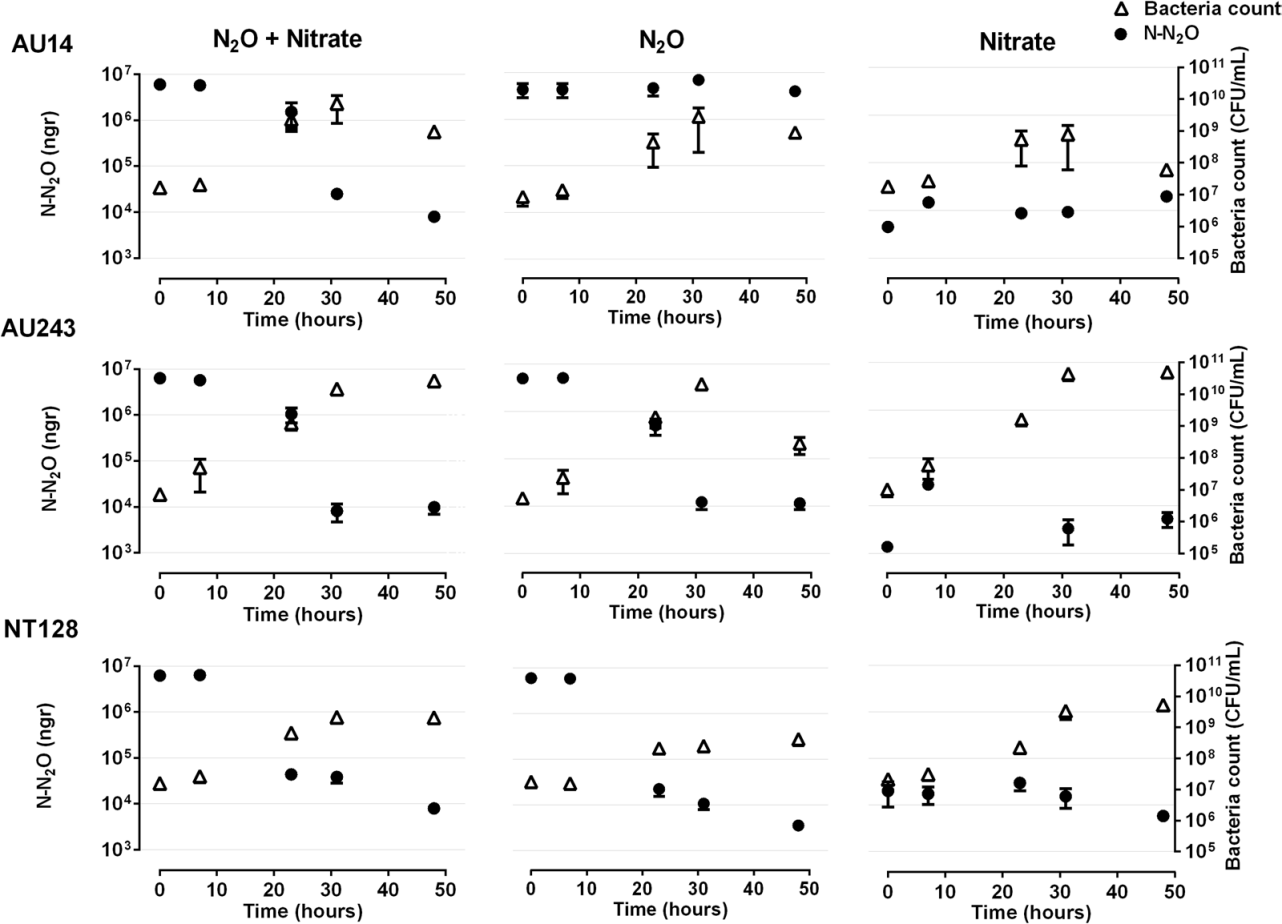
N_2_O reduction by isolates AU14, AU243 and NT128 and their growth in the presence of N_2_O, nitrate or both, with acetate, arabinose and galactose as carbon sources. Error bars depict standard errors (n = 4).

Denitrifiers possessing *nosZ* have been shown to reduce N_2_O with different efficiencies^27^. Many studies have used acetylene as an inhibitor of N_2_O reduction to measure N_2_O-reduction efficiency in pure culture^30,31^. However, the results of such measurements can be overestimates, as acetylene also inhibits several N_2_O-producing processes^32^. Therefore, we tested the actual ability of the isolates to reduce N_2_O in aqueous media using GC analysis of the residual N_2_O over time in media with different compositions: with or without nitrate in the presence of N_2_O and acetate (Fig. S1), or all three carbon sources (acetate, arabinose and galactose, Fig. 2). In media containing either acetate alone or a mixture of the three carbon sources, as well as both nitrate and N_2_O, the three isolates behaved similarly, and were able to reduce all available N_2_O (ca. 6300 gr N-N_2_O), (Figs S1, 2).

Interactions between nitrogen and carbon sources were observed in some of the media, where differences between the isolates were observed. For example, in the presence of N_2_O alone, isolate AU14 partially reduced N_2_O, when the three carbon sources were supplied (Fig. 2). However, when only acetate was used as the carbon source, isolate AU14 was able to completely reduce N_2_O at rates similar to those of the two other isolates (Fig. S1). This isolate cannot use galactose or arabinose as sole carbon source (data not shown), and their presence seems to inhibit its N_2_O-reduction rate.

In the presence of nitrate alone (no N_2_O addition), N_2_O did not accumulate in the headspace of any of the cultures (Fig. 2). Isolates AU243 and NT128 were able to reduce nitrate, as indicated by the consumption of TN (consisting mainly of nitrate; Fig. S2) and consequently, were able to reduce the produced N_2_O to atmospheric nitrogen. Isolate AU14 behaved differently: it did not change the nitrate level, due to its inability to reduce it (Fig. S2), and thus N_2_O was not produced and no N_2_O accumulation was observed (Fig. 2).

The three isolates showed a pronounced ability to reduce N_2_O, even in the presence of nitrate in the media (Fig. 2). Isolate NT128 reduction rate was ca. 132 gr N-N_2_O/h, followed by isolates AU243 and AU14 (ca. 131 and 125 gr N-N_2_O/h, respectively). Interestingly, although isolate AU243 was the least efficient N_2_O reducer, it utilized twice the amount of carbon and grew 1.5- to 2-fold more than the other two isolates within 50 h in both media with nitrate addition (Fig. S2). As for isolate AU14, although it lacks the nitrate reductase genes (*Nar* and *Nap*; Fig. 1), N_2_O reduction was more efficient in the presence of nitrate (Fig. 2; N_2_O+ nitrate), possibly due to its use as a nitrogen source for catabolism. The ability of the tested isolates to reduce total N_2_O in the presence of nitrate suggests that they have the potential to reduce N_2_O to atmospheric nitrogen in soil fertilized with nitrate, and thus may be used to mitigate N_2_O emissions. In the next experiment, we therefore inoculated wheat plants and measured possible N_2_O mitigation in a plant–soil mesocosm.

### Dynamics of wheat root colonization

**Figure 3:**
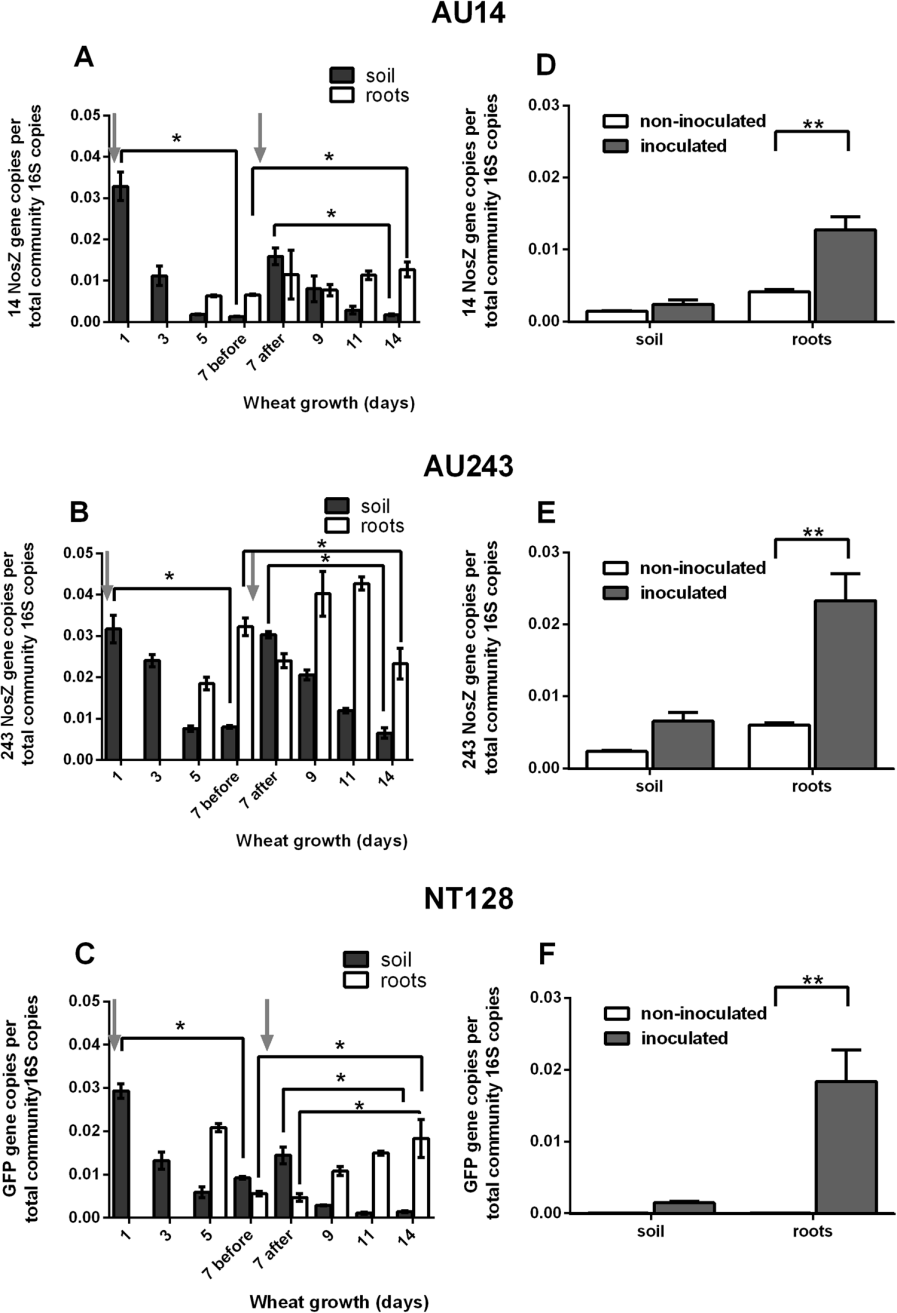
Population dynamics of the N_2_O-reducing isolates in soil and on roots following inoculation. (**A**–**C**) Relative abundance of N_2_O-reducing isolates AU14, AU243 and NT128, respectively, in soil (black) and wheat roots (white) during 14 days of wheat growth. Bacteria were inoculated at seeding and on day 7 of wheat growth (marked with arrows). (**D**–**F**) Relative abundance of AU14, AU243 and NT128, respectively, in inoculated (white) vs. non-inoculated (black) samples after 14 days of wheat growth. Significance in difference between day 1–7 before inoculation and between 14 and 7 (before or after inoculation) is marked with * (Table S2). **Significant enrichment (*p* < 0.001) in the roots inoculated with bacteria compared to non-inoculated controls. Error bars depict standard errors (n = 3).

Real-time PCR quantification of root and rhizosphere colonization is much more feasible and less time-consuming than the dilution-plate counting method, especially when dealing with aggregating bacteria, and it can be performed long after sampling. Moreover, using strain-specific PCR quantification is much more accurate and can work in various niches containing endogenous bacterial populations, some of which are highly similar morphologically^33^. Here, efficiency of wheat root colonization and survival of the three isolates inoculated in soil were monitored over a period of 14 days. The total bacterial community measured as 16S gene copy number was between ~8.7E6 and 1.6E7 per gr soil and 1.4E6 to 6.75E6 per gr roots during 14 days in inoculated and non-inoculated treatments. Isolates were quantified in the soil and roots using real-time PCR measurement of *nosZ* (isolates AU14 and AU243) or GFP (isolate NT128) (Table S2) and normalized to 16S rDNA copies (Fig. 3). Genome analysis of each isolate have shown that each genome contains only one copy of nitrous-oxide reductase (EC 1.7.99.6) based on SEED and RAST. At initial soil inoculation, before seed planting, the amounts of each of the isolates were comparable at around 0.03 gene copies per bacterial 16S copy (Fig. 3A–C). One week after inoculation, the relative abundance of all isolates in the soil decreased significantly. After a second soil inoculation on day 7, their relative abundance increased, followed by a second gradual decrease. Minor and transient effects of soil inoculation on the native community agrees with previous studies on crop plant inoculation with plant-growth-promoting or biocontrol bacterial agents (reviewed by Minz *et al*.^34^).

A sufficient amount of root material for genomic DNA extraction could only be obtained from day 5 of wheat growth onward. During the week following second inoculation, root colonization levels of the isolates were at around 1% of total 16S (for isolate AU14), 2–4% (for AU243) and 1–2% (for NT128) (Fig. 3A–C).

The inoculation of isolates AU14 and AU243 was successful, with relative abundance on the roots that was 3.1 and 3.9 times higher than in natural, non-inoculated soils, respectively (note that each was amplified with specific *nosZ* primers). Isolate AU128, however, was amplified with GFP-specific primers and thus non-inoculated roots showed no signal. The bacterial DNA copy number per gram of root was around 10^4^ for each of the isolates (calculated from data in Fig. 3). Soil inoculation with each of the three isolates resulted in a non-significant increase in abundance after 14 days (Fig. 3D–F). All three bacteria, originally isolated from wheat roots, successfully colonized the root zone rather than the surrounding soil (Fig. 3A–C). It is plausible that the denitrification traits of those isolates is responsible for this success in root colonization^35^.

### Bacterial inoculation may affect N_2_O emissions from wheat mesocosms

**Figure 4:**
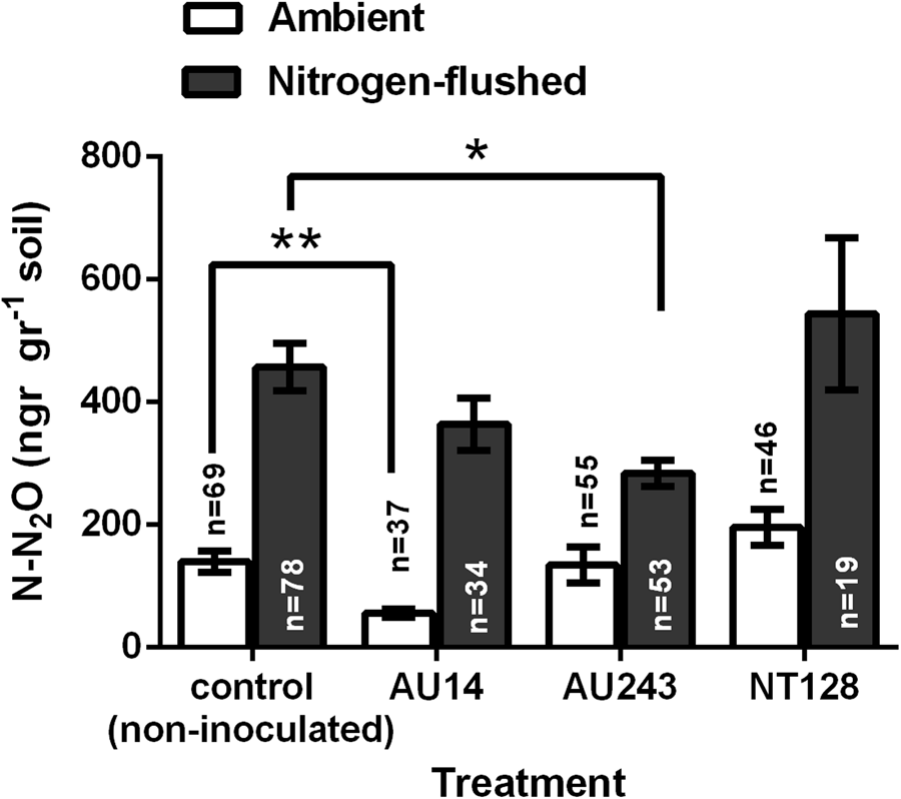
Effect of inoculation with isolates AU14, AU243 and NT128 on N_2_O emission from wheat mesocosms. N_2_O emission in ambient (white) and nitrogen-flushed (black) atmosphere was monitored from wheat mesocosms with and without (control) inoculation. The experiment was repeated multiple independent times with different mesocosm, with total number of replicates marked in the bars. Error bars depict standard error means (SEM). *, **Significant decrease in N_2_O emissions from non-inoculated controls at *p* = 0.0183 and *p* = 0.0018, respectively.

Cereal fields cover around 53% of the crop-growing area worldwide and require continuous growth (www.fao.org/faostat/en/#data/QC)^8^. These fields are the major source of anthropogenic N_2_O emissions^5^. Here we suggest the possibility of using N_2_O-reducing bacteria for mitigation of N_2_O emissions from such soils. We tested the ability of the three N_2_O-reducing, wheat root-colonizing isolates to mitigate N_2_O emission from a mesocosm containing 14-day-old wheat plants. Initially, N_2_O emissions were measured from plants growing in pots placed in 1-L glass jars with di-nitrogen (N_2_)-enriched atmosphere to reduce oxygen content, and to promote denitrification in the soil. Plants were kept in the closed jars for 10 h of light, followed by 12 h of darkness prior to N_2_O measurement. Net N_2_O emission plausibly varied during incubation and after 22 h of incubation, net N_2_O emission of ca. 457 ng N-N_2_O gr^−1^ soil was measured in the non-inoculated control mesocosm. Inoculation of isolates AU14 and AU243 resulted in an up to 20% and 38% decrease in N_2_O emissions, respectively, compared to the control, whereas isolate NT128 enhanced N_2_O emissions by 19% (Fig. 4).

Similarly, N_2_O emission was measured in ambient, non-manipulated atmosphere, in separated set of jars and individual plants. Under these conditions, closing non-inoculated mesocosms in jars resulted in a N_2_O level of 140 ng N-N_2_O gr^−1^ soil, significantly lower than the level in the nitrogen-flushed atmosphere, as expected. Inoculation with isolate AU14 decreased N_2_O emissions by up to 60%, whereas isolate AU243 did not decrease N_2_O emission compared to the control, and isolate NT128 enhanced N_2_O emissions by 39% (Fig. 4).

All three isolates were able to reduce N_2_O in liquid media and efficiently colonized wheat roots. However, they showed different patterns of N_2_O-emission reduction in the mesocosm. The rhizospheric soil is characterized by varying aerobic/anoxic niches that are constantly changing because of varying carbon concentrations, and water content affecting oxygen availability, as well as respiration of the roots and associated microorganisms^36,37^. Two representative sets of conditions were tested in the current study. The first set included reduced oxygen levels by altering the pot atmosphere through di-nitrogen flushing, forming partly anoxic conditions, which then fluctuated slightly with some oxygen supply from photosynthesis, followed by its consumption during the light–dark cycle. The second set, under ambient conditions, was achieved by closing the jar lid and creating conditions as close to ambient as possible during the light–dark cycle. Thus, the ability of the three studied isolates to affect N_2_O emission was tested under two very different conditions. Although each isolate was able to reduce N_2_O efficiently in pure liquid culture, under these conditions, with each isolate in an inoculation pot, the results differed. Isolate AU243 caused a decrease in N_2_O emission under lower but not ambient oxygen conditions. Under both conditions, however, isolate AU14 decreased N_2_O emission, whereas isolate NT128 showed slightly increased emissions. The latter may be the result of NT128’s ability to reduce nitrate in the plant–soil system under the studied conditions. It was previously shown that by the addition of non-denitrifying strain of *Dyadobacter fermentans* harbouring clade II nosZ, it was possible to considerably mitigate N_2_O emissions in more than third of the studied soils^38^. Moreover, to the best of our knowledge, only two published studies have reported the use of N_2_O-reducing bacteria to mitigate N_2_O emissions in soil-grown plants. Gao *et al*.^19^ tested inoculation of N_2_O-reducing denitrifiers *Azospirillum* and *Herbaspirillum* spp. in pasture soil under greenhouse conditions, while Itakura *et al*.^20^ used the symbiotic nitrogen-fixing *Bradyrhizobium japonicum* to inoculate soybean growing in the field. In contrast, we isolated and tested free-living root bacteria with an affinity for wheat roots, and potentially other cereals^25^.

We demonstrated the potential of bacterial inoculants to mitigate N_2_O emission from the rhizosphere. Our results showed that the genetic ability to reduce N_2_O in pure culture cannot predict the actual *in situ* activity when inoculated into a soil–plant system. All tested isolates, when inoculated into the soil–plant system, preferred to colonize the roots. However, each isolate operates differently at diverse environmental conditions e.g. altered oxygen levels. For example, as isolate AU243 is able to reduce both nitrate and N_2_O it may operate better at lower oxygen levels. On the other hand, isolate AU14, lacking the nitrate reductases, might operate better under less anoxic conditions based only on N_2_O reduction, by itself or provided by other bacteria. Although performed in pots with one specific soil and plant species, our results emphasize the importance of a deep understanding of soil–plant–microbe interactions when environmental application is being considered.

## Materials and Methods

### Isolation of N_2_O-reducing bacteria

Bacteria were isolated from wheat roots by the following enrichment procedure. Roots of wheat plants at early tillering stage, grown for 10 days in agricultural soil, were washed in saline solution and placed in 100-mL flasks. Each flask contained 10 mL medium designed for enrichment of N_2_O-reducing bacteria^24^ without NO_3_^−^ addition. Enrichment medium also contained 16 mM acetate, 8.6 mM galactose, 8.6 mM arabinose and 2 mL trace elements added to 1 L solution (134 mM NaEDTA, 22 mM ZnSO_4_·7H_2_O, 50 mM CaCl_2_, 22 mM MnSO_4_·H_2_O, 18.5 mM FeSO_4_·7H_2_O, 1 mM (NH_4_)_2_MoO_4_, 4.3 mM CuCl_2_·2H_2_O, 65 μM CoCl_2_). Flasks were sealed with a rubber folding stopper, allowing for gas sample injection and collection. Flasks were adjusted to anaerobic conditions by flushing for 5 min with 2 atm nitrogen (99.99%), and 20 mL N_2_O gas was added as the terminal electron acceptor, using a polypropylene syringe. After several enrichment steps under these conditions, bacteria were isolated on petri dishes without nitrate and incubated in a desiccator under anaerobic conditions (flushed for 2 min with 1 atm nitrogen and an additional flushing with 1 atm N_2_O gas for 1 min). In the next step, selected isolates were streaked on petri dishes with or without a nitrate source to test their ability to reduce nitrate as well as N_2_O under N_2_O atmosphere. The isolates that grew better in the presence of N_2_O were selected for N_2_O-reduction test under liquid conditions.

### Bacterial DNA extraction

Bacteria were grown for 17 h in 4 mL LB medium and total genomic DNA was extracted using the Exgene Cell SV DNA isolation kit (GeneAll, Seoul, Korea) according to the manufacturer’s instructions. DNA yield and quality were examined with an ND1000 spectrophotometer (NanoDrop Technologies, Wilmington, DE), and agarose gel electrophoresis.

### Preparation of libraries, sequencing and annotation

Isolates genomic DNA was prepared for shotgun metagenome sequencing using the Nextera XT DNA Library Preparation Kit (Illumina, San Diego, CA) at the DNA Services Facility of the University of Illinois at Chicago. Library was sequenced on an Illumina NextSeq500, employing paired-end 150 base reads. In addition, Nanopore libraries were constructed utilizing the Oxford Nanopore genomic DNA library protocol SQK-NSK007, according to the manufacturer’s instructions (Oxford Nanopore Technologies), and sequencing was performed using a FLO-MIN104 (R9 Version) flow cell on an Oxford Nanopore MinION instrument. A total of 3,963,710 and 27,058,816 sequences were obtained from the Illumina MiSEq, and a complimentary 3,558 and 13,992 from the Nanopore sequencing for isolate AU14 and AU243 respectively. De novo assembly was performed using the Spades assembler^39^, with multiple k-mers specified as “-k 27, 47, 67”. Coverage levels were assessed by mapping raw Illumina reads to the contigs with bowtie2^40^, and computing coverage as the number of reads aligning per contig, times length of each read, divided by length of the contig. Contigs were then filtered by coverage level: contigs were first sorted by coverage, and a coverage threshold was taken as 1/2 of the contig coverage at 50% of the total assembly length. To get functional annotation for the genes, assembled reads were analysed using SEED and the Rapid Annotation of microbial genomes using Subsystems Technology (RAST). Whole isolates genome sequences have been submitted to the National Centre for Biotechnology Information (NCBI) databases under Accession Number: SAMN09841847 and SAMN09841848 under the BioProject ID: PRJNA486231.

### N_2_O-Reduction test in pure culture

N_2_O-reduction test was used to evaluate the overall ability of each isolate to reduce nitrate, N_2_O, or both, as well as the reduction rates. Isolates were grown overnight in 4 mL LB media, washed in sterile saline, resuspended in 4 mL fresh saline and a 0.1-mL aliquot was then injected into a 10-mL glass vial sealed with an aluminum crimp cap with butyl rubber septum (La-Pha-Pack, Langerwehe, Germany). Each vial contained 4 mL N_2_O medium with nitrate (36 mM KNO_3_) adjusted to anaerobic conditions by flushing for 2 min with 2 atm nitrogen and addition of 0.5 mL N_2_O gas. N_2_O concentration in the atmosphere was analyzed after 48 h of growth by gas chromatography (GC) (450-GC Greenhouse Analyzer, Varian, Middelburg, the Netherlands) with a headspace auto sampler (Teledyne Tekmar, Mason, OH) and electron-capture detector. N_2_O concentrations were corrected to N_2_O solubility in the aqueous phase using the Bunsen solubility coefficient^41^, representing total N_2_O concentrations (ppm) in the headspace and medium. After initial screening, the most promising isolates were tested by growing overnight in 4 mL LB media, washed in sterile saline, resuspended in 4 mL fresh saline and a 0.5-mL aliquot was then injected into 200-mL glass bottles. Those bottles were sealed with an aluminum crimp cap, containing 50 mL N_2_O media with nitrate (36 mM KNO_3_), adjusted to anaerobic conditions by flushing for 2 min with 2 atm nitrogen followed by addition of 22 mL N_2_O gas. The following parameters were measured periodically during 48 h of bacterial growth: N_2_O concentration, bacterial counts (CFU), total soluble organic carbon (TOC), total soluble nitrogen (TN), and soluble ammonia and nitrate. The medium was filtered (0.45 µm) and TOC was measured by TOC-V_CPN_ (Shimadzu, Kyoto, Japan), equipped with a total nitrogen measuring unit (TNM-1 Shimadzu) for TN, and ammonia and nitrate were measured with a Gallery™ Plus Automated Photometric Analyzer (Thermo Fisher Scientific, Waltham, MA).

### Plant growth and bacterial inoculation

Wheat (*Triticum turgidum* cv. Negev) was cultivated in dune sand mixed with clay soil obtained from non planted-soil margins of wheat field in Volcani center, Rishon Le-Zion, Israel, in order to get sandy clay loam. The soil was air-dried, passed through a 2.0-mm sieve and stored at room temperature (~25 °C) prior to the experiments. Soil parameters, total soluble organic carbon (TOC), total soluble nitrogen (TN), and soluble ammonia, nitrate and phosphate were measured (Table 1) as described above.

**Table 1:** Parameters of soil used for cultivating wheat.

Parameters	Values
soil structure	sandy clay loam
Sand (%)	60.5
Silt (%)	12.1
Clay (%)	27.4
pH	7.9 ± 0.1
EC (µS/m)	168 ± 7
N-NO_3_ (gr/kg)	0.28 ± 0.03
NH_4_ (gr/kg)	0.18 ± 0.00
P-PO_4_ (gr/kg)	0.04 ± 0.00
TOC (gr/kg)	2.0 ± 0.4
TC (gr/kg)	3.3 ± 0.4
IC (gr/kg)	1.3 ± 0.1
TN (gr/kg)	0.6 ± 0.1

Seeds were surface-sterilized by soaking in 3% sodium hypochlorite for 30 s followed by 70% ethanol for 45 s and washed with water. Soil was distributed into 300-mL plastic pots, 250 g each, and four seeds were planted per pot. Prior to inoculation, isolates AU243, NT128 and AU14 were grown for 72 h in N_2_O isolation medium, centrifuged at 2700 *g* for 10 min, and suspended in modified Hoagland nutrient solution^25^ with 3.6 mM KNO_3_ added to 1 L solution. 50 ml of this nutrient solution was used for pots irrigation three times a week for two weeks. Pots were inoculated twice by irrigation (on the day of seed planting and 1 week later) to achieve high root colonization levels. The plants were grown in a growth chamber maintained at 22 °C with 12 h of daylight for 2 weeks, after which N_2_O emission from the pots was measured. Pots were not irrigated 72 hours prior to N_2_O emission measurements. Each plant mesocosm was destructively sampled following one 22 h incubation period. Up to 13 individual experiments were conducted with 4–6 replicates per treatment, the statistical analyzes was conducted combining all replicates.

### N_2_O emission from wheat mesocosm

To test N_2_O emissions, the 2 weeks old plants in pots were placed in 1-L glass jars that were tightly sealed with a silicone ring and high-vacuum grease (DOW Corning, Midland, MI) equipped with penetrable septa for gas sampling. N_2_O emission was measured under two conditions: (a) di-nitrogen(N_2_)-enriched atmosphere: air was flushed out by 2 atm nitrogen over a period of 2 min, and (b) ambient atmosphere: no gas exchange. Jars were stored in a plant growth chamber under a 12 h light/12 h dark regime for 22 h. Gas samples were collected once at the end of 22 h of incubation by inserting a polypropylene syringe needle through the septum in the jar top and slowly withdrawing 0.1 mL and 1 mL for nitrogen-enriched and ambient atmosphere measurements, respectively. Samples were immediately transferred to 20-mL glass vials sealed with an aluminum crimp cap containing a butyl rubber septum and analyzed by GC as described above.

### Soil and root DNA extraction for qPCR

At each sampling time point, individual pots were randomly selected for DNA extraction, while the remaining pots were used for N_2_O emission tests. Soil and wheat roots were collected (in triplicate) every 2 days for 2 weeks. Total DNA was extracted from 0.4 g roots or 0.3 g soil, using the Exgene Soil DNA mini isolation kit (GeneAll) according to the manufacturer’s instructions.

### Generation of quantitative PCR plasmid standards

NosZ sequences of the isolates were compared to nosZ sequences in NCBI and FunGene (http://fungene.cme.msu.edu/) databases. Primers were designed to fit sequence where no identical ones were in these databases (Table 2). The partial *nosZ* sequence from isolates AU14 and AU243 was generated by PCR T100™ Thermal Cycler (Bio-Rad, Hercules, CA) using the PCR primers NosZ14-1720F/14R and NosZ-243F/R respectively (Table 2). Partial green fluorescent protein (GFP) and 16S rDNA sequences were generated as described previously^28,42^. Each *nosZ* PCR amplification product was ligated into pGEM-T Easy Vector (Promega, Madison, WI). The plasmids were transformed into BioSuper *Escherichia coli* DH5α competent cells (Bio-lab, Jerusalem, Israel). Linearized plasmid DNA (*nosZ* gene of isolate AU243) and circular plasmid DNA (*nosZ* gene of isolate AU14, GFP and 16S) were used as standards to create calibration curves in 10-fold dilutions for gene quantification by RT- PCR.

**Table 2:** Primers Used for Quantitative PCR.

Target gene	Target isolate	Primer name	Primer sequence (5’ → 3’)	Primer usage	Amplicon size (bp)	Reference
nosZ	AU14	nosZ14-823F	GGG AAT AGA TGT AGG CGA GAT GAG C	RT-PCR target	135	this study
		nosZ14-958R	AAA AGG CCT ACG ACC TGG GC			
		nosZ14-1720F	TTA ACT CCG GTG GTC AAT CC	Quantification plasmid	1706	this study
		nosZ-14R	CAG GCT TTG GGT TTC ACA TT			
	AU243	nosZ243-790F	AGG AAG GCG TGAA CCT CCA G	RT-PCR target	134	this study
		nosZ243-924R	AAC TTC GAG CCG TGG CGA			
		nosZ-243F	GAT CCG TAT TGT CGG TCT GC	Quantification plasmid	1529	this study
		nosZ-243R	TGA TGA GAA GCC GTG AG			
GFP	NT128	GFP_F	CACTGGAGTTGTCCCAATTC	RT-PCR target	150	Tovi *et al*.^28^
		GFP_R	GGC CAT GGA ACA GGT AGT TT			
16S	Total bacterial community	16S-331F	TCC TAC GGG AGG CAG CAG T	RT-PCR target	195	Hunter *et al*.^43^ Lopez *et al*.^44^
		16S-518R	ATT ACC GCG GCT GCTG G			

### Quantitative PCR assessment of gene copy numbers

Dynamics of bacterial movement from the soil to the wheat roots over 2 weeks of wheat growth was monitored by following *nosZ* gene copy number using a StepOnePlus Real-Time PCR System (Applied Biosystems, Foster City, CA). Triplicates of extracted DNA were diluted to 6 ng/µL and 1 µL was used in a final reaction volume of 20 µL together with 50 µM forward and reverse primers and 10 µL Fast SYBR MasterMix (Thermo Fisher Scientific). Total bacterial abundance was estimated by targeting the 16S rDNA gene using universal primers 16S-331F/518R (Table 2). Samples were denatured at 95 °C for 5 min, followed by 40 cycles at 95 °C for 5 s and 60 °C for 30 s. Three technical replicates were conducted for each individual soil or root DNA sample. Reaction efficiency was monitored in each run by means of an internal standard curve (constructed plasmids) using duplicates of 10-fold dilutions of standards ranging from 10^8^–10^2^ copies per reaction. Reported efficiency was 95%–100% for all target genes and runs, and R^2^ values were greater than 0.99. Copy number of target gene (e.g. NosZ, GFP and 16S) was calculated based on calibration curve of plasmid copy number. All data analyses were conducted using StepOne software v2.3 (Applied Biosystems). Relative abundance of N_2_O-reducing isolates was calculated by dividing specific target gene (e.g. 14 NosZ, 243 NosZ or GFP) copy number by copy number of 16S gene, which represents total bacteria community in the sample.

### Statistical analysis

Statistical analysis was performed by non-parametric comparisons with control using Steel’s method or comparisons for each pair using Wilcoxon method in JMP 13 Pro (SAS Institute Inc., Cary, NC) and statistical significance was set at *p* < 0.05.

## Supplementary information

Supporting information
