# Supplementary material for: Altering N2O emissions by manipulating wheat root bacterial community
Source: Sci Rep. 2019 May 20;9:7613. doi: 10.1038/s41598-019-44124-3 (PMC6527579; doi:10.1038/s41598-019-44124-3)
Supplement: Supplementary file 1 — Supporting information [file 41598_2019_44124_MOESM1_ESM.pdf]

Altering N_2O emissions by manipulating wheat root bacterial community

Alla Usyskin-Tonne,[1,2] Yitzhak Hadar,[2] and Dror Minz[1,*]

[a]Soil, Water and Environmental Sciences, Agricultural Research Organization, Volcani Center, Rishon LeZion, Israel

[b]Robert H. Smith Faculty of Agriculture, Food and Environment, The Hebrew University of Jerusalem, Rehovot, Israel

*minz@volcani.agri.gov.il

Supporting information

Table S1. Amount of nosZ gene in inoculated pots with isolates AU243 and AU14 and amount of GFP gene in inoculated pots with isolate NT128, respectively, and non-inoculated (control) samples during 14 days of wheat growth.

week	inoculation	Mean nosZ or GFP copy number per gr soil	Standard error	Mean nosZ or GFP copy number per gr root	Standard error
1	NT128	3.2E+05	1.9E+04	NA	NA
3		2.1E+05	3.5E+04	NA	NA
5		7.0E+04	1.6E+04	4.1E+04	6.5E+02
7 before		9.8E+04	2.1E+04	4.5E+04	1.5E+04
7 after		1.6E+05	2.3E+04	1.2E+04	4.2E+03
9		2.5E+04	6.4E+03	2.4E+04	2.4E+03
11		6.4E+04	2.5E+04	2.3E+04	4.6E+03
14		7.2E+03	1.5E+03	2.0E+04	3.2E+03
14	Control GFP	2.1E+01	2.8E+00	6.6E+01	1.6E+01
1	AU243	2.7E+05	2.5E+04	NA	NA
3		1.7E+05	1.0E+04	NA	NA
5		5.8E+04	7.9E+03	4.9E+04	5.4E+03
7 before		1.0E+05	3.6E+03	7.3E+04	6.4E+03
7 after		3.6E+05	2.6E+04	4.3E+04	3.1E+03
9		2.5E+05	1.2E+04	7.2E+04	2.6E+03
11		8.7E+04	1.4E+04	8.8E+04	8.4E+03
14		6.8E+04	1.2E+04	6.3E+04	4.9E+03
1	AU14	3.6E+05	4.1E+04	NA	NA
3		1.2E+05	2.9E+04	NA	NA
5		2.3E+04	1.4E+03	2.1E+04	3.7E+02
7 before		2.3E+04	2.5E+03	2.3E+04	4.1E+02
7 after		2.9E+05	4.1E+04	3.0E+04	2.3E+03
9		1.1E+05	6.1E+04	2.9E+04	5.8E+03
11		5.3E+04	1.7E+04	2.2E+04	1.8E+02
14		2.8E+04	2.0E+03	1.8E+04	6.3E+02
14	Control nosZ	2.6E+04	1.3E+03	1.5E+04	6.0E+02

NA- not available, roots were sampled starting from 5 day.

Table S2. Non-parametric comparisons for each week pair using Wilcoxon method for RT-PCR measurements.

Selected isolates		AU14		AU243		NT128	
Compared pairs (weeks)		**Soil** (p-Value)	**Roots** (p-Value)	**Soil** (p-Value)	**Roots** (p-Value)	**Soil** (p-Value)	**Roots** (p-Value)
1	3	0.0015	NA	0.2893	NA	0.0018	NA
1	5	0.0004	NA	0.0004	NA	0.0004	NA
1	7 before	0.0004	NA	0.0004	NA	0.0018	NA
1	7 after	0.002	NA	0.4799	NA	0.0008	NA
1	9	0.0027	NA	0.0091	NA	0.0018	NA
1	11	0.0004	NA	0.0004	NA	0.0018	NA
1	14	0.0001	NA	<0.0001	NA	<0.0001	NA
1	14 control	<0.0001	NA	0.0001	NA	0.001	NA
3	5	0.0004	NA	0.0004	NA	0.0292	NA
3	7 before	0.0004	NA	0.0004	NA	0.1735	NA
3	7 after	0.0772	NA	0.002	NA	0.7683	NA
3	9	0.4437	NA	0.0772	NA	0.0051	NA
3	11	0.0036	NA	0.0004	NA	0.0051	NA
3	14	0.0001	NA	<0.0001	NA	0.0004	NA
3	14 control	<0.0001	NA	0.0001	NA	0.0034	NA
5	7 before	0.0027	0.2664	0.0004	0.0237	0.0062	0.0282
5	7 after	0.0004	0.4595	1	0.1551	0.2629	0.0282
5	9	0.3165	0.1106	0.0004	0.0332	0.1407	0.0162
5	11	0.5365	0.0645	0.0015	0.0162	0.0018	0.0282
5	14	0.3028	0.1235	0.0001	0.9497	0.0002	0.1296
5	14 control	0.0087	0.0182	0.0605	0.0077	0.001	0.0081
7 before	7 after	0.0004	0.3311	0.0004	0.0048	0.0875	0.5752
7 before	9	0.316	0.0198	0.0004	0.1426	0.0051	0.0056
7 before	11	0.2508	0.0062	0.0006	0.0031	0.0051	0.0051
7 before	14	0.2408	0.02	0.0069	0.0185	0.0004	0.0057
7 before	14 control	0.797	0.0003	0.0001	<0.0001	0.0034	0.0004
7 after	9	0.0872	0.5966	0.0004	0.0426	0.0018	0.0027
7 after	11	0.0004	0.0849	0.0004	<0.0001	0.0018	0.0051
7 after	14	0.0001	0.0838	<0.0001	0.23	<0.0001	0.0032
7 after	14 control	<0.0001	0.817	0.0001	<0.0001	0.001	0.0004
9	11	0.1753	0.0262	0.0004	0.8598	0.0051	0.0183
9	14	0.2814	0.2045	0.0001	0.0074	0.0057	0.241
9	14 control	0.2432	0.0071	0.0001	<0.0001	0.0034	<0.0001
11	14	0.4996	1	0.0059	0.0089	0.5059	0.4824
11	14 control	0.2267	<0.0001	0.0001	<0.0001	0.0034	0.0004
14	14 control	0.3095	<0.0001	0.0012	<0.0001	0.0002	<0.0001

NA- not available, roots were sampled starting from 5 day.

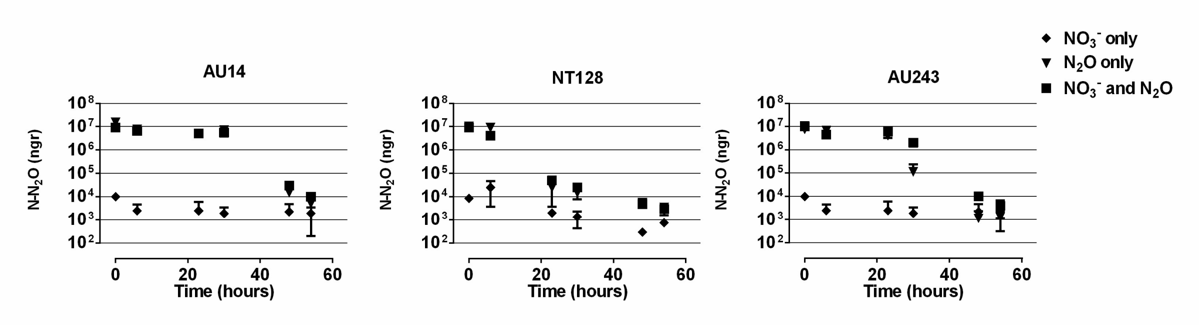

Figure S1. N_2O reduction by isolates AU14, AU243 and NT128 in the presence of N_2O, nitrate or both, with acetate as carbon source. Error bars depict standard errors (n = 2).

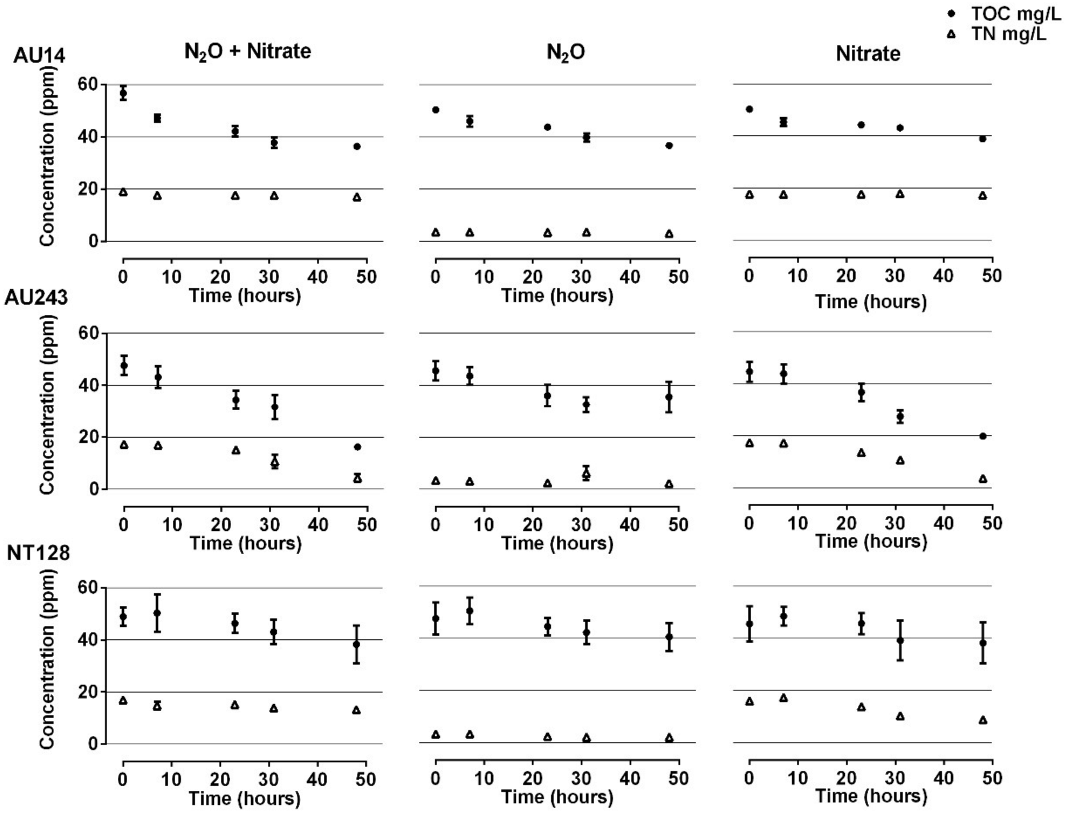

Figure S2. Total soluble organic carbon and nitrogen contents (TOC and TN, respectively) in media during growth of isolates AU14, AU243 and NT128 in the presence of N_2O, nitrate or both. Error bars depict standard errors (n = 4).
